# Atmospheric Neutron Monitoring through Optical Fiber-Based Sensing

**DOI:** 10.3390/s20164510

**Published:** 2020-08-12

**Authors:** Sylvain Girard, Adriana Morana, Cornelia Hoehr, Michael Trinczek, Jeoffray Vidalot, Philippe Paillet, Camille Bélanger-Champagne, Julien Mekki, Nicolas Balcon, Gaetano Li Vecchi, Cosimo Campanella, Damien Lambert, Emmanuel Marin, Aziz Boukenter, Youcef Ouerdane, Ewart Blackmore

**Affiliations:** 1 Univ Lyon, UJM, CNRS, IOGS, Laboratoire Hubert Curien, UMR 5516, 18 rue Prof. B. Lauras, 42000 Saint-Etienne, France; adriana.morana@univ-st-etienne.fr (A.M.); jeoffray.vidalot@univ-st-etienne.fr (J.V.); gaetano.li.vecchi@cern.ch (G.L.V.); cosimo.campanella@univ-st-etienne.fr (C.C.); emmanuel.marin@univ-st-etienne.fr (E.M.); aziz.boukenter@univ-st-etienne.fr (A.B.); ouerdane@univ-st-etienne.fr (Y.O.); 2 TRIUMF, 4004 Wesbrook Mall, Vancouver, BC V6T 2A3, Canada; choehr@triumf.ca (C.H.); trinczek@triumf.ca (M.T.); cbchampagne@triumf.ca (C.B.-C.); ewb@triumf.ca (E.B.); 3 CEA, DAM, DIF, F-91297 Arpajon, France; philippe.paillet@cea.fr (P.P.); damien.lambert@cea.fr (D.L.); 4 CNES, 18 avenue Edouard Belin, 31400 Toulouse, France; Julien.Mekki@cnes.fr (J.M.); Nicolas.Balcon@cnes.fr (N.B.); 5 European Organization for Nuclear Research (CERN), CH-1211 Geneva, Switzerland

**Keywords:** radiation monitor, fiber sensors, atmospheric neutron, dosimetry, optical fibers, radiation effects, space, optical time domain reflectometry

## Abstract

The potential of fiber-based sensors to monitor the fluence of atmospheric neutrons is evaluated through accelerated tests at the TRIUMF Neutron Facility (TNF) (BC, Canada), offering a flux approximatively 10^9^ higher than the reference spectrum observed under standard conditions in New York City, USA. The radiation-induced attenuation (RIA) at 1625 nm of a phosphorus-doped radiation sensitive optical fiber is shown to linearly increase with neutron fluence, allowing an in situ and easy monitoring of the neutron flux and fluence at this facility. Furthermore, our experiments show that the fiber response remains sensitive to the ionization processes, at least up to a fluence of 7.1 × 10^11^ n cm^−^², as its radiation sensitivity coefficient (~3.36 dB km^−1^ Gy^−1^) under neutron exposure remains very similar to the one measured under X-rays (~3.8 dB km^−1^ Gy^−1^) at the same wavelength. The presented results open the way to the development of a point-like or even a distributed dosimeter for natural or man-made neutron-rich environments. The feasibility to measure the dose caused by the neutron exposure during stratospheric balloon experiments, or during outer space missions, is presented as a case study of a potential future application.

## 1. Introduction

Cosmic rays from deep space and high-energy solar particles strike the Earth’s upper atmosphere, where they interact with its oxygen and nitrogen atoms to produce particle cascades of secondary radiation. These cascades lead to a variety of particles, such as protons, neutrons or pions; the atmospheric neutrons are the final end product of all these interactions [1]. The atmospheric neutron flux depends on the altitude, as a result of the competition between those cascade effects and the atmosphere shielding effect, since both increase with the crossed atmosphere thickness [2]. The maximum neutron flux (~10^4^ n cm^−2^ h^−1^) occurs at an altitude of ~20 km (~60,000 ft) and decreases at lower altitude, the flux being on the order of ~10^3^ n cm^−2^ h^−1^ at an airplane’s altitude (~40,000 ft), and ~10 n cm^−2^ h^−1^ at sea level [3]. Monitoring these atmospheric neutrons is of major importance, since it has been shown that they are able to cause single event effects (SEEs) in modern micro-electronic technologies, affecting the performance and reliability of modern highly-integrated devices operating in altitude or even at the Earth’s surface [4]. SEEs (see [5] for a review) are caused by the energy deposited in the device by a single neutron. If this energy deposition results in a charge collection exceeding the critical charge of the technology used, different SEEs are susceptible to occur [5]. To evaluate the vulnerability of a given technology, or the efficiency of mitigation techniques against atmospheric neutron events, accelerated tests have to be performed by the radiation effects community. To this purpose, dedicated facilities, such as the Los Alamos Neutron Science Center (LANSCE) [6] or TRIUMF [7] provide a high flux of atmospheric neutrons thanks to a spallation source, or by using a 400 MeV proton beam impinging upon an aluminum target, respectively. As a consequence, there is a strong need for devices and systems to allow the monitoring of the atmospheric neutron flux or fluence, either to characterize the different natural environments associated with aerospace applications or to enhance the reliability of the SEE radiation tests performed at facilities. As an example, researchers have demonstrated the potential of monitoring SEEs in calibrated Static Random Access Memories (SRAMs) to map the proton or neutron fluence during radiation tests [8]. In this paper, we demonstrate the highly promising potential of optical-fiber sensing, to monitor the atmospheric neutrons. It is already known that the monitoring of the radiation-induced degradation in the fiber through either radiation-induced attenuation (RIA) or radiation-induced emission (RIE) phenomena [9,10] could be exploited to design radiation detectors or dosimeters. We studied a solution that relies on the RIA measurement through optical time domain reflectometry (OTDR) of a radiation sensitive optical fiber exposed to very high flux of atmospheric neutrons at the TRIUMF facility. As an application case study, the feasibility of using fiber sensors in the future to monitor the neutron fluence during stratospheric balloon experiments is investigated on the basis of the acquired experimental data.

## 2. Materials and Methods

**Figure 1:**
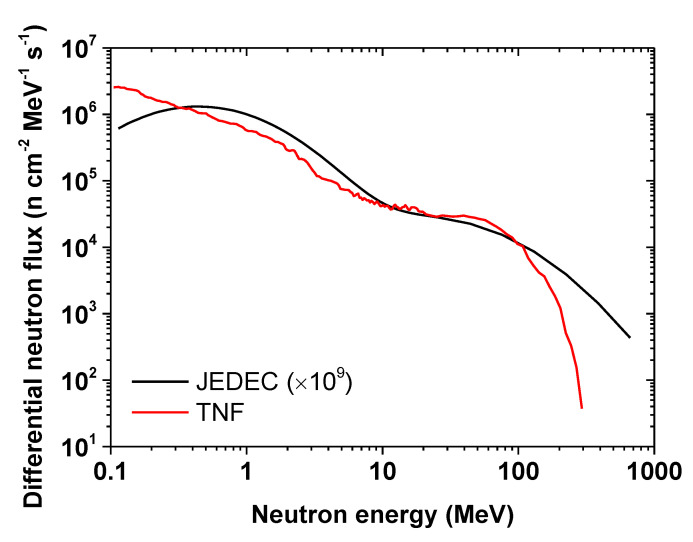
Simulated neutron spectrum of the TRIUMF TNF facility, compared to the JEDEC atmospheric neutron reference spectrum. Accelerated tests were possible at a flux approximatively 10^9^ times higher than on the Earth’s ground.

The experiments were conducted at the TRIUMF Neutron Facility (TNF) facility in Vancouver, Canada, where a 480 MeV proton beam is employed for experiments in the high-intensity beam line, BL1A. After traversing several experiments, the protons of 100 to 150 µA current and 400 to 450 MeV residual energy were stopped in a beam dump on an aluminum plate absorber surrounded by a water moderator [7]. Neutrons were produced from spallation reactions and were subsequently channeled through the shielding surrounding the TNF. The resulting neutron spectrum, simulated with FLUKA (CERN, Geneva, Switzerland), is illustrated in Figure 1 and compared to the Joint Electron Device Engineering Council (JEDEC) standard JESD89A of terrestrial cosmic rays, which induces soft errors in semiconductor devices [11]. This comparison shows that the TNF facility is well adapted for the accelerated testing of the vulnerability of technologies to these radiation constraints, offering a flux approximatively 10^9^ higher than the reference spectrum observed under standard conditions in New York City, USA. The devices to be tested to neutron exposure at the TNF were mounted on an aluminum plate and lowered about 5 m into the neutron beam through a cable system in a vertical access channel. Once the device (a fiber coil, in our case) was in place, a counter recorded the neutron exposure in a neutron detector at beam level. The selected optical fiber was exposed in June 2019, at room temperature (RT), to an integrated neutron flux of ~3.35 × 10^6^ n [10–400 MeV] cm^−2^ s^−1^ for approximately 59 h to reach a total fluence of ~7.1 × 10^11^ n cm^−^². The FLUKA simulations of the TNF neutron spectrum (Figure 1) were exploited, in order to calculate the equivalent dose and dose rate in Gy(SiO_2_) and Gy/s associated with these test conditions. From these calculations, which considered neutrons within an energy spectrum from 0.1 to 400 MeV, a relationship was obtained between the neutron fluence and the equivalent dose in Gy(SiO_2_). The neutron run was associated with an equivalent dose rate of ~0.91 Gy/h, up to a total dose of ~53.8 Gy(SiO_2_). The irradiation temperature remained between 20 °C and 30 °C.

**Figure 2:**
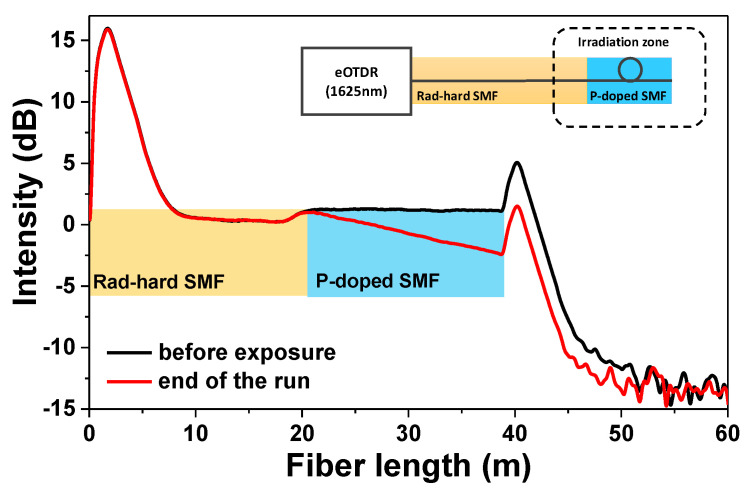
eOTDR traces measured at 1625 nm before and at the end of the neutron irradiation (total fluence of ~7.1 × 10^11^ n cm^−2^). Inset: schematic of the experimental setup used for the RIA measurement.

About 18 m of a P-doped single-mode fiber (SMF) from iXBlue (Lannion, France) (see [12,13] for a more complete description of this radiosensitive fiber) was coiled with a 50 mm diameter, a size compatible with the beam homogeneity at TNF. This fiber coil was spliced to a ~20 m pigtail of radiation-hard F-doped SMF from iXblue to enable the signal transport from the neutron-beam zone to the instrumentation zone, where the interrogator, an embedded optical time domain reflectometer (eOTDR) from Viavi Solutions, was located. The eOTDR sent light pulses at 1625 nm through the radiation-hard fiber to the P-doped SMF and measured all along the fiber line the intensities of the Rayleigh backscattered light at the same wavelength. As the SMF is exposed to the radiation, the backscattered light decreases over time, due to radiation-induced attenuation in the radiosensitive SMF. A schematic of the setup is illustrated in the Figure 2 inset. This figure also illustrates the typical OTDR traces acquired before and after irradiation, at a probe wavelength of 1625 nm. The pulse width duration was set to 10 ns (allowing a one-meter spatial resolution), and one measurement point was performed every 4 cm. During the neutron run, one OTDR trace was acquired every minute.

## 3. Results

**Figure 3:**
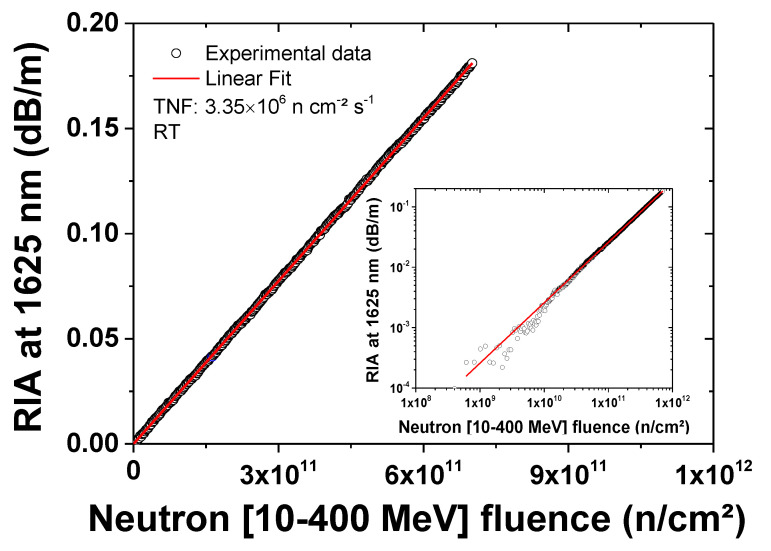
RIA growth at 1625 nm in an 18 m long coil of the P-doped fiber exposed to the TNF neutron [10–400 MeV] fluence at room temperature (RT). In the inset the same results are illustrated in a log-log scale.

From the acquired OTDR traces, the radiation-induced optical losses (RIA at 1625 nm) can be extracted, and their evolution, with the increase in neutron fluence, can be investigated. This is shown in Figure 3, which illustrates the RIA evolution at 1625 nm during the neutron exposure for the P-doped single-mode fiber, at an average flux of 3.35 × 10^6^ n cm^−2^ s^−1^. The induced losses evolve linearly with the deposited fluence (integrated between 10 and 400 MeV by the TRIUMF monitor count) at a rate of ~6.8 × 10^−4^ dB m^−1^ (n^−1^ cm^2^). Looking at the inset of Figure 3, we estimate that, by using an 18 m long P-doped fiber, it is possible to detect fluences above 10^9^ n cm^−2^ with this setup (it should be hereby considered that some of the beam-flux fluctuations, shown in the inset of Figure 3, occurred during the first hours of our tests, and were due to beam instabilities during the 520 MeV cyclotron operations). These results confirm the potential of a radiosensitive fiber to monitor the neutron integrated fluence in real-time, even in complex environments with wide neutron-energy spectra, as in the case of atmospheric neutrons. If a reflectometer is used to measure the optical losses, such sensors should also be able to offer spatially resolved measurements. However, only sufficiently high losses (corresponding to a quite high fluence) can be detected when a one-meter (typical for OTDR) resolution is achieved.

## 4. Discussion

**Figure 4:**
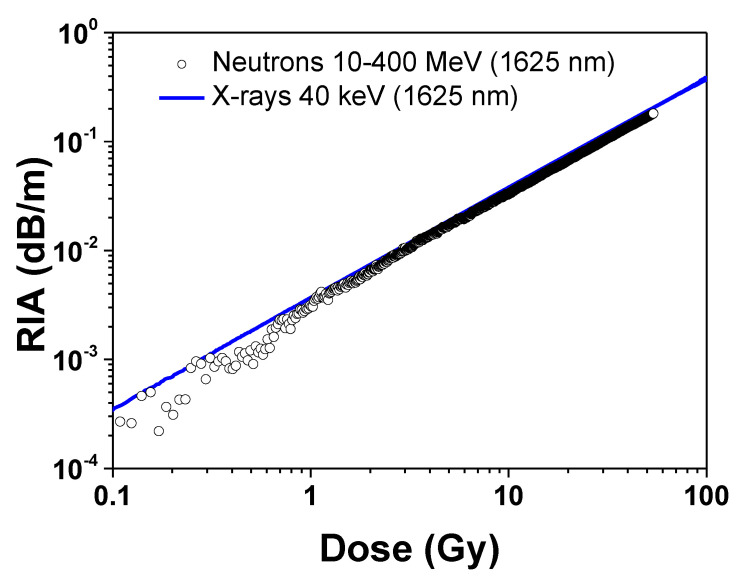
Comparison between the dose dependence of the RIA, induced at 1625 nm by 40 keV X-rays and atmospheric neutrons in a phosphorus-doped single-mode optical fiber.

Several previous studies have shown that the main process leading to the generation of point defects in silica-based optical fibers is ionization, whereas displacement damage only marginally contributes to the generation of point defects, except at very high neutron fluences (>10^16^ n cm^−2^) [10,14,15]. In the case of P-doped fibers, it has been shown that the infrared optical losses are similar for equivalent doses (usually calculated with Geant4 or FLUKA Monte-Carlo packages) of X-rays, γ-rays, protons or the complex mixed environments of the CHARM facility, a Proton Synchrotron and Proton Synchrotron Booster at CERN [16,17,18]. It is then possible to measure the ionizing dose with the optical fiber, regardless of the complexity of the considered environments in terms of a particle’s nature. For this experiment, and thanks to the FLUKA simulation of the TNF facility, we determined the 1625 nm RIA dependence versus the equivalent dose deposited by the atmospheric neutrons and verified the ability of the fiber to act as a dosimeter in the TNF facility. It should be noted that the neutron capacity to ionize silica is only indirect, i.e., via the creation of secondary particles through nuclear reactions. These secondary particles interact with the atomic electrons and ionize them [19]. The RIA dose dependence is illustrated in Figure 4 and compared to the one measured at the same wavelength under X-rays (at a dose rate of 15.5 Gy/h, average photon energy of 40 keV) from the MOPERIX facility of the LabHC, France. For the neutron tests, we found a radiation sensitivity coefficient at 1625 nm of ~3.36 dB km^−1^ Gy^−1^ for this fiber (the fit standard error and Adj. R-Square are of 2.4 × 10^−4^ and 0.99998, respectively) to be very close to the coefficient obtained at the same wavelength under X-rays: 3.80 dB km^−1^ Gy^−1^ (the fit standard error and Adj. R-Square of 2.2 × 10^−4^ and 0.99992, respectively). The small difference between those coefficients cannot be discussed, as it remains within our experimental and theoretical uncertainties. This demonstrates the ability of the fiber sensor to monitor the equivalent dose, associated with the atmospheric neutrons.

These results also confirm the previous studies and show that the point defect at the origin of the 1625 nm RIA, the P1 defect [20], is mainly created through ionization processes, while displacement damage only marginally contributes to the induced losses at the considered fluences. The P1 defect is associated with one absorption band peaking at around 1.6 µm (0.79 eV with a full width at half maximum (FWHM) of 0.29 eV), very close to the chosen eOTDR wavelength [20]. This is one of the specificities of the fiber radiation responses, compared to most of the other opto- or micro-electronic technologies, for which ionization and displacement damage usually lead to clearly distinguishable effects [21]. This means that for such fiber-based sensors, the calibration done under X-rays or γ-rays is sufficient to operate in mixed environments, including different particles or radiation at different energies, greatly simplifying their application, and widening their potential application. The sensor will provide information about the Total Ionizing Dose, often called TID. If combined with Monte-Carlo simulations of the mixed environments, or with other detector technologies giving the expected energy distribution of particles, the results provided by the sensor could also help determine the particle flux and fluence.

**Figure 5:**
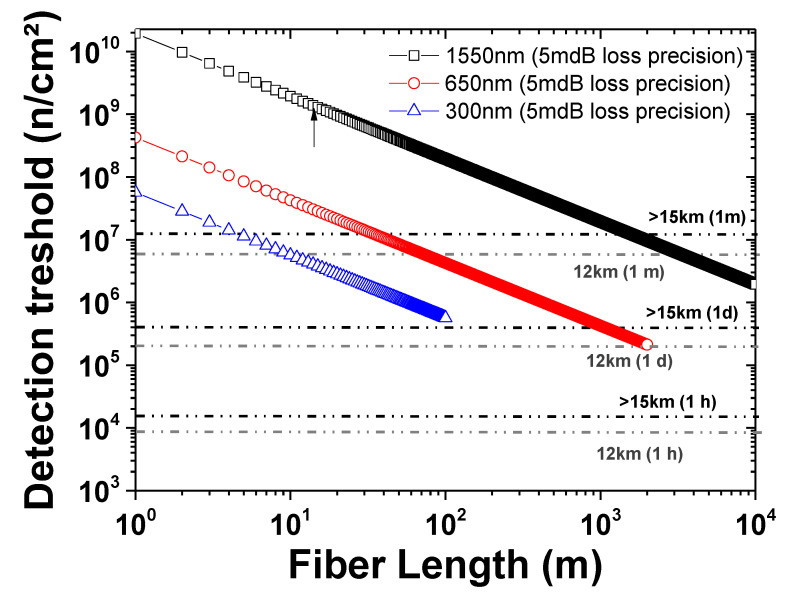
Comparison between the fluence detection thresholds of fiber sensors operating at either around 1550 to 1625 nm, in the UV (around 300 nm) or in the visible (around 650 nm). The horizontal lines indicate the expected equivalent doses, depending on the flight parameters (altitude, duration: 1 hour (h), 1 day (d), 1 month (m)).

From the acquired data, extrapolations can be made to evaluate the potential of fiber sensing for man-made (radiation facilities) or natural (atmosphere, space…) environments. We investigate here the feasibility of monitoring the neutron flux with a fiber sensor, during stratospheric balloon experiments. Such experiments are used as a platform to investigate the upper atmosphere, for environmental, or weather characterization or to assess new technologies for aerospace needs [22]. For these calculations, we considered the different profiles of balloon experiments, both in terms of altitude (12 km or above 15 km with the highest neutron flux), and in terms of flight duration (from 1 h to 1 month). This allowed for the determination of the expected neutron fluences in these different operation conditions, which are reported in Figure 5. This figure compares the expected fluences with the performance achievable, via fiber sensing through RIA, using various sensor architectures.

Here, we also consider the different parameters that can be adjusted to optimize the sensor detection threshold. First, the sensitive fiber length can easily be increased, automatically decreasing the neutron fluence detection threshold. The limitation to this approach arises from the fiber’s intrinsic losses at the probe wavelength: here 10 dB of intrinsic losses and 20 dB of intrinsic losses were considered as manageable for distributed OTDR-based sensor and point sensor (laser diode as a source and a photodiode as the detector), respectively. At 1550 nm, with a 10 km fiber coil and assuming that a RIA around ~5 mdB can be achieved with an optimized system, the minimal detectable fluence should be about 10^6^ to 2 × 10^6^ n cm^−^², which is typically reached in about one week, at altitudes higher than 15 km. It should also be considered that the temperature range during balloon experiments (−60 °C to RT) is quite large, and that, id2ature dependence of the IR-RIA in P-doped fibers at low doses, the preliminary results at higher doses and the dose rate would reveal that the 1550 nm RIA levels are comparable (within 20%) between −40 °C and RT [23]. Another possibility to change the detection threshold is to shift the probe wavelength from 1550 nm to the ultraviolet (UV) or visible domains, where the fiber is more radiation sensitive. Indeed, it has been shown that even if the point defects at the origin of the RIA will be different than at 1550 nm (P1 defects [20]), the P-doped fibers still present some interesting dosimetry properties at around 300 nm (where the P2 defect contribution is preponderant [20,24,25]), and at around 650 nm (where the phosphorus-oxygen hole centers (POHC) defects are the main contributors [20,24,25]), with RIA kinetics being dose rate and temperature (0 to 50 °C) independent at low doses (<100 Gy). The P2 defects are associated with an absorption band that peaked at 275 nm (4.5 eV, FWHM = 1.27 eV), whereas POHC have absorption bands at 563 nm (2.2 eV, FWHM = 0.35 eV) and 496 nm (2.5 eV, FWHM = 0.63). At these two wavelengths, the fibers are more radiation sensitive than at 1550 nm (~3.8 dB km^−1^ Gy^−1^) with sensitivity coefficients around 1000 dB km^−1^ Gy^−1^ and 100 dB km^−1^ Gy^−1^, respectively [24]. Furthermore, a previous study showed that X-rays, γ-rays and 14 MeV neutrons lead to similar RIA levels in the visible domain as well, when normalized to the deposited dose [26]. These results justify our hypothesis that, as for P1 defects in the IR domain, atmospheric neutrons will probably mainly create the P2 and POHC defects through ionizing processes. However, we should also note that at these two wavelengths, the fiber intrinsic losses increase to about 10 dB km^−1^ at 650 nm and 200 dB km^−1^ at 300 nm, limiting the lengths of the fiber coil to 2 km and 100 m, respectively. Considering this limitation, our analysis shows that working in the UV domain will probably not allow the fluence detection threshold to be decreased. Optimized sensors operating in the visible should allow an in-situ monitoring of the dose, related to the atmospheric neutrons (and the neutron fluence if combined with radiation environment simulations) on a daily basis at altitudes higher than 15 km.

## 5. Conclusions

We characterized the radiation-induced attenuation (RIA) of a phosphorus-doped single-mode optical fiber when exposed to a high flux neutron environment (atmospheric spectrum of the TRIUMF TNF facility). We showed that its attenuation at 1625 nm linearly increases with neutron fluence, allowing in situ and real-time monitoring of the neutron fluence at least up to a fluence of 7.1 × 10^11^ n cm^−2^. Through FLUKA simulations of the TNF environment and the dose deposited by the neutrons in the silica-based optical fiber, we observed that the fiber radiation sensitivity coefficient (~3.4 dB km^−1^ Gy^−1^) under neutron exposure was very close to that measured under X-rays (~3.8 dB km^−1^ Gy^−1^) at the same wavelength. The acquired results allow us to evaluate the potential of point or distributed-dose measurements in various natural or man-made neutron-rich environments. For the application case study of stratospheric balloon experiments, monitoring the neutron fluence during experiments with a duration longer than one week appears achievable using the radiation-sensitive fiber and the monitoring of its radiation-induced losses, either in the infrared (around 1550 nm) or the visible (around 650 nm) domains. Future studies are still needed to better assess the temperature dependence of the phosphorus-doped fiber radiation sensitivity coefficient in the range encountered by balloon experiments (−60 to 30 °C), and its impact on the radiation detection.
